# Efficacy and Safety of Obturator Nerve Block in Transurethral Resection of Bladder Tumors: A Comprehensive Review

**DOI:** 10.7759/cureus.65859

**Published:** 2024-07-31

**Authors:** Shubham Rahane, Neeta Verma

**Affiliations:** 1 Anaesthesiology, Jawaharlal Nehru Medical College, Datta Meghe Institute of Higher Education and Research, Wardha, IND

**Keywords:** surgical complications, bladder cancer, obturator reflex, anesthesia techniques, obturator nerve block (onb), transurethral resection of bladder tumors (turbt)

## Abstract

Transurethral resection of bladder tumors (TURBT) is a pivotal procedure in the management of bladder cancer, essential for both diagnosis and treatment. Effective anesthesia is crucial in TURBT to ensure a stable and pain-free operative field, facilitate precise tumor resection, and minimize complications such as the obturator reflex, which can lead to involuntary leg movement and bladder injury. The obturator nerve block (ONB) is a regional anesthesia technique designed to prevent the obturator reflex by blocking the obturator nerve, which innervates the adductor muscles of the thigh. This comprehensive review evaluates the efficacy and safety of ONB in TURBT. It begins by discussing the anatomical and physiological aspects of the obturator nerve, followed by a detailed examination of various ONB techniques, including ultrasound-guided and landmark-based methods. The review assesses the impact of ONB on pain management, reduction of adductor muscle spasms, and overall improvement in surgical conditions and patient satisfaction. Additionally, it explores the incidence and types of complications associated with ONB, such as hematoma, nerve injury, and local anesthetic systemic toxicity (LAST). It compares ONB with other anesthesia techniques used in TURBT, such as general, spinal, and epidural anesthesia. A critical analysis of key clinical studies and meta-analyses is presented to provide a comprehensive understanding of the current evidence on ONB efficacy and safety. Future directions and innovations in ONB techniques, including advances in imaging and nerve localization, are also discussed. Practical recommendations for implementing ONB in clinical practice, including guidelines for clinician training and patient selection criteria, are provided. This review aims to inform clinicians about the benefits and risks of ONB in TURBT, guide clinical practice, and identify areas for future research to optimize anesthesia management in bladder cancer surgery.

## Introduction and background

Transurethral resection of bladder tumors (TURBT) is a cornerstone in the management of bladder cancer. This endoscopic procedure involves the resection of visible tumors within the bladder through the urethra using a resectoscope [1]. TURBT serves diagnostic and therapeutic purposes, enabling histopathological evaluation and local tumor control. Bladder cancer is the tenth most common cancer worldwide, with approximately 573,000 new cases and 213,000 deaths annually. TURBT is crucial for staging bladder cancer, determining muscle invasiveness, and guiding subsequent treatment strategies, including intravesical therapy, systemic chemotherapy, or radical cystectomy [2]. Effective anesthesia is vital for the success of TURBT. The procedure requires a stable and pain-free operative field to facilitate precise tumor resection and minimize patient discomfort and movement. Anesthesia management in TURBT aims to provide adequate pain relief, muscle relaxation, and hemodynamic stability [3]. Furthermore, anesthesia techniques should minimize the risk of complications, such as bladder perforation or obturator reflex, which can lead to involuntary leg movement and pose a significant challenge to surgeons. Various anesthesia options, including general, spinal, epidural, and regional nerve blocks, are employed to meet these objectives [4].

The obturator nerve block (ONB) is a regional anesthesia technique to prevent the obturator reflex during TURBT. The obturator nerve, originating from the lumbar plexus (L2-L4), innervates the adductor muscles of the thigh [5]. Stimulation of this nerve during bladder tumor resection can lead to sudden adductor muscle contraction, risking bladder wall injury or perforation. ONB involves the injection of a local anesthetic around the obturator nerve, effectively blocking motor and sensory transmission. This technique enhances surgical conditions by preventing involuntary muscle contractions and improving patient safety [6]. This comprehensive review aims to evaluate the efficacy and safety of obturator nerve blocks in TURBT. It will explore the anatomical and physiological basis of the obturator nerve, various ONB techniques, and their impact on surgical outcomes. The review will also compare ONB with other anesthesia modalities, discuss potential complications, and provide practical recommendations for clinical practice. By synthesizing current evidence and identifying gaps in knowledge, this review seeks to inform clinicians and guide future research on optimizing anesthesia management for TURBT.

## Review

Anatomy and physiology of the obturator nerve

The obturator nerve is a vital structure from the lumbar plexus, specifically from the anterior rami of spinal nerves L2, L3, and L4. Its anatomical pathway begins in the lumbar plexus, formed by the ventral divisions of these lumbar spinal nerves [7]. After its formation, the nerve descends through the fibers of the psoas major muscle, emerging from its medial border near the pelvic brim. Continuing its course, the obturator nerve runs posterior to the common iliac arteries and lateral to the internal iliac vessels before exiting the pelvis through the obturator canal, located in the obturator foramen - a bony opening in the pelvis [7]. Upon entering the medial compartment of the thigh, the obturator nerve divides into two main branches: the anterior branch and the posterior branch [8]. The anterior branch passes deep to the adductor longus muscle and innervates the adductor longus, brevis, gracilis, and occasionally the pectineus muscle. Additionally, it provides sensory innervation to the skin over the proximal part of the medial thigh [7]. In contrast, the posterior branch pierces the obturator externus muscle, supplies the adductor magnus and obturator externus, and provides articular branches to the knee joint. This dual role of the obturator nerve - motor and sensory - highlights its importance in lower limb function [7]. In terms of clinical relevance, particularly in the context of TURBT, the obturator nerve's anatomical proximity to the surgical field is significant [9]. During TURBT, inadvertent stimulation of the obturator nerve can lead to involuntary contractions of the adductor muscles, complicating the surgical procedure. To mitigate this risk, an obturator nerve block is often performed [10]. This block effectively prevents complications associated with nerve stimulation, such as adductor muscle spasms and bladder perforation, thereby enhancing surgical safety and improving patient comfort. Overall, understanding the anatomy and physiology of the obturator nerve is essential for urologists and surgeons to optimize outcomes during procedures involving the bladder and pelvic region [10].

Techniques of obturator nerve block

The ultrasound-guided technique has gained popularity due to its precision and effectiveness. This method can be executed through either a proximal or distal approach. In the proximal approach, a single local anesthetic injection is administered into the interfascial plane between the pectineus and obturator externus muscles [11]. This targets both the anterior and posterior branches of the obturator nerve, often resulting in a higher success rate in blocking the nerve than distal approaches [12]. The proximal technique may also reduce the required dose of local anesthetic, providing better analgesia, particularly for hip-related procedures. In the distal approach, the ultrasound transducer is placed at the inguinal crease, and local anesthetic is injected into the interfascial planes between the adductor muscles. Typically, two injections are given: one between the adductor longus and brevis (anterior branch) and another between the adductor brevis and magnus (posterior branch). While effective, this method may have a lower success rate than the proximal approach [12]. The landmark-based technique, on the other hand, relies on anatomical landmarks without imaging guidance. This traditional method involves locating the obturator nerve based on its relationship to surrounding structures, such as the pubic bone and adductor muscles. The classic landmark approach can utilize the pubic or inguinal methods, where the needle is inserted at specific points to target the obturator nerve [13]. Although this method has been used for many years, it is less precise than ultrasound-guided techniques and may result in variable success rates due to anatomical variations among patients [13].

**Figure 1:**
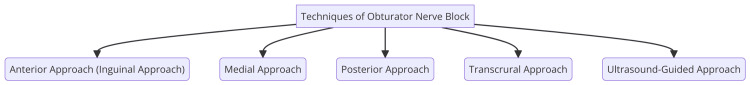
Techniques of obturator nerve block Image Credit: Dr. Shubham Rahane

When comparing these techniques, several factors include efficacy, safety, and procedure time. Studies have demonstrated that ultrasound-guided techniques, particularly the proximal approach, yield a higher success rate in achieving adequate local anesthetic spread into the obturator canal than landmark-based techniques [14]. For instance, one study reported an 84% success rate for the ultrasound-guided method, while the pubic and inguinal approaches showed only 42.6% and 47.8%, respectively [14]. Regarding safety, ultrasound guidance significantly reduces the risk of complications such as vascular puncture and nerve injury by allowing real-time visualization of anatomical structures. In contrast, landmark-based techniques carry a higher risk of complications due to their reliance on palpation and anatomical knowledge alone [15]. Additionally, while ultrasound-guided techniques may require more initial setup time, they can lead to quicker and more efficient procedures overall, as their higher success rates often reduce the time spent on the block [15]. The obturator nerve block is primarily indicated during TURBT [3]. One of the main indications is the prevention of the obturator reflex, which can result in involuntary thigh adduction during the procedure, potentially leading to complications such as bladder perforation. Additionally, an obturator nerve block provides effective analgesia, enhancing patient comfort during and after the procedure, particularly for patients undergoing extensive resections [3]. Furthermore, an obturator nerve block is often utilized as an adjunct to other anesthesia techniques, such as spinal or general anesthesia, to improve overall pain control and reduce the need for systemic analgesics. By addressing these factors, the obturator nerve block plays a crucial role in ensuring the safety and efficacy of TURBT procedures [16]. Techniques for obturator nerve block are shown in Figure 1.

Efficacy of obturator nerve block

The ONB has emerged as a valuable technique in managing pain and improving surgical outcomes during TURBT [12]. Effective pain management is crucial for this procedure, as TURBT can be associated with significant discomfort. When ONB is combined with spinal anesthesia, it alleviates pain and enhances the overall patient experience [6]. Studies have shown that patients undergoing TURBT with ONB report lower pain scores and greater satisfaction compared to those who do not receive this nerve block. This combination allows for a more comfortable surgical experience, which is essential for patient well-being and surgical efficacy [17]. One of the primary challenges during TURBT is the risk of adductor muscle spasms, which can occur due to stimulation of the obturator nerve. These spasms can lead to complications such as bladder perforation and incomplete tumor resection. The implementation of ONB has significantly reduced the incidence of these spasms. Techniques such as the nerve locator method and ultrasound-guided ONB have successfully prevented adductor reflexes [18]. For instance, studies indicate that the nerve locator technique can effectively reduce the occurrence of adductor spasms. In contrast, ultrasound-guided ONB with nerve stimulation has achieved up to 90% success rates. This reduction in muscle spasms improves surgical conditions and allows for a more efficient and uninterrupted procedure [18].

In addition to pain management and muscle spasm reduction, ONB contributes to overall improvements in surgical conditions. Surgeons have reported enhanced visibility and access to the surgical field when ONB is utilized, which is critical for successfully resectioning bladder tumors [19]. The combination of reduced muscle activity and improved analgesia allows for a more controlled surgical environment, facilitating better outcomes. Furthermore, patients and surgeons have expressed high satisfaction with using ONB during TURBT, highlighting its role in enhancing the surgical experience [19]. Clinical outcomes associated with ONB are generally favorable. The incidence of bladder perforation remains low, and adverse events related to the nerve block are infrequent when performed by experienced practitioners [3]. The integration of ONB with spinal anesthesia not only minimizes complications but also contributes to a smoother recovery process for patients. Overall, the use of ONB in TURBT has been linked to improved clinical outcomes, including reduced postoperative pain and quicker recovery times, further emphasizing its importance in this surgical context [3].

Safety and complications

ONB is generally a safe procedure, but it is not without potential complications. Among the most common issues associated with ONB are hematoma formation, nerve injury, and local anesthetic systemic toxicity (LAST). Hematomas can occur if blood vessels are accidentally punctured during the injection process, leading to localized swelling and discomfort [3]. Nerve injury, although rare, can result from incorrect needle placement or direct trauma to the nerve, potentially causing temporary or, in very rare cases, permanent dysfunction. LAST is a serious complication that arises from intravascular injection or excessive dosage of a local anesthetic, leading to symptoms such as seizures, cardiovascular instability, and respiratory distress. Immediate treatment is crucial and typically involves the administration of intravenous lipid emulsion and supportive care [3]. The incidence of complications during TURBT when utilizing ONB is relatively low. Studies have shown that using ONB significantly reduces the risk of bladder perforation and obturator nerve reflex [20]. For instance, the rate of bladder perforation is reported to be around 11% when ONB is employed, compared to a much higher rate of 53% in procedures without ONB. Similarly, the incidence of the obturator nerve reflex drops from 46% to 22% with this nerve block. These statistics highlight the effectiveness of ONB in enhancing patient safety during TURBT [20]. Several strategies can be implemented to minimize the risks associated with ONB during TURBT. First and foremost, employing proper technique is essential; performing ONB under ultrasound guidance combined with nerve stimulation can significantly improve the accuracy of needle placement, thereby reducing the likelihood of complications. Additionally, careful dosing of local anesthetics is critical to prevent LAST; the dosage should be tailored to the patient's weight and overall health status [20]. Continuous patient monitoring during and after the procedure is also vital for the early detection and management of adverse effects. Finally, selecting the specific ONB technique based on the individual patient's condition, tumor location, and the surgeon's experience can optimize both efficacy and safety. By adhering to these strategies, healthcare providers can enhance the safety profile of ONB during TURBT, leading to better outcomes for patients [20].

Comparison with other anesthetic techniques

The choice between general anesthesia and ONB for TURBT depends on the specific benefits and drawbacks of each technique. General anesthesia provides complete unconsciousness and muscle relaxation, which is beneficial for extensive surgical procedures. However, it is associated with higher risks of systemic complications, such as respiratory issues, and prolonged recovery times [3]. Additionally, in TURBT, general anesthesia alone may not effectively prevent obturator nerve reflexes, leading to potential complications like bladder perforation. In contrast, ONB specifically targets the obturator nerve to prevent involuntary adductor muscle contractions during TURBT. Studies indicate that ONB significantly reduces the incidence of obturator reflexes compared to general anesthesia alone, making it a valuable adjunct in TURBT procedures [3]. When comparing spinal and epidural anesthesia to ONB, both techniques effectively provide analgesia and muscle relaxation during TURBT. However, spinal anesthesia alone does not adequately prevent obturator nerve reflexes, which can lead to complications during surgery. The combination of spinal anesthesia with ONB has been shown to significantly lower the incidence of obturator reflex (11.4% with ONB vs. 40% without) and improve overall surgical outcomes. ONB enhances the effectiveness of spinal anesthesia by specifically addressing the risks associated with obturator nerve reflexes. When used alongside spinal anesthesia, ONB can lead to better control of intraoperative conditions and reduce complications related to adductor muscle spasms [17]. Each anesthetic technique has its benefits and drawbacks. General anesthesia provides complete control over patient awareness and muscle relaxation, making it suitable for extensive procedures, but it carries higher risks of systemic complications and longer recovery times [21]. Spinal anesthesia is effective for pain control and muscle relaxation. Still, it may be inadequate in preventing obturator reflex without ONB, and it can lead to potential complications such as hypotension and post-dural puncture headache. ONB specifically prevents obturator nerve reflex and adductor spasms, and it can be combined with other anesthetic techniques for enhanced safety. However, it requires skill and experience to perform effectively and carries a potential risk of localized complications such as vascular puncture [21].

Review of clinical studies and meta-analyses

Several key clinical trials have investigated the efficacy and safety of ONB in TURBT. One notable study compared bipolar TURBT with traditional monopolar techniques and found that bipolar TURBT resulted in less postoperative hemoglobin decline and shorter catheterization duration. Specifically, the mean postoperative change in hemoglobin was significantly lower in the bipolar group (−0.58 g/dL vs. −0.95 g/dL, P = 0.038), and the duration of catheterization was reduced (2.20 days vs. 2.65 days, P = 0.026). These findings suggest that bipolar TURBT may offer improved safety and recovery profiles [22]. In terms of ONB efficacy, a systematic review highlighted its role in preventing the obturator nerve reflex (ONR) during TURBT. The analysis revealed that adding ONB to spinal anesthesia significantly reduced the incidence of ONR and bladder perforation, with a risk reduction ratio (RR) of 0.22 for both complications when ONB was employed. This underscores the importance of ONB as a valuable adjunct in enhancing surgical outcomes. Additionally, a meta-analysis demonstrated that enhanced techniques, such as blue light cystoscopy (BLC), detected significantly more Ta/T1 tumors and carcinoma in situ (CIS) lesions compared to white light cystoscopy (WLC). This suggests that integrating advanced imaging techniques can further improve surgical outcomes in TURBT [17].

Meta-analyses have provided robust evidence regarding the efficacy and safety of ONB in TURBT. A systematic review of randomized controlled trials evaluating ONB indicated a high success rate in preventing ONR, with studies reporting success rates between 93% and 100%. The analysis concluded that ONB is a safe and effective adjunct to spinal anesthesia, significantly reducing intraoperative complications. Furthermore, comparative studies found that ONB improved intraoperative conditions by minimizing involuntary muscle contractions, leading to better surgical outcomes and reduced complication rates [23]. The safety profile of ONB was consistently favorable across various studies, with minimal adverse effects reported. Most complications associated with ONB were transient and included minor motor weakness at the injection site, which resolved quickly. This positive safety profile reinforces the recommendation for ONB in TURBT procedures, particularly for patients at higher risk for complications [24]. The findings from clinical trials and meta-analyses underscore the critical role of ONB in enhancing the safety and efficacy of TURBT. The significant reduction in ONR and bladder perforation rates when ONB is utilized suggests that this technique should be considered a standard practice in TURBT procedures. Moreover, the comparison between bipolar and monopolar TURBT techniques reveals that bipolar TURBT not only minimizes intraoperative risks but also improves postoperative recovery metrics [17]. Additionally, the enhanced detection rates of tumors with advanced imaging techniques like BLC further support the need for integrating these methods into routine practice to optimize patient outcomes. Integrating ONB and advanced surgical techniques in TURBT represents a significant advancement in urological surgery, providing a framework for improved patient safety and surgical effectiveness. Future research should continue exploring the long-term outcomes of these techniques to solidify their roles in clinical practice [22].

Future directions and innovations

Recent imaging technology advancements have significantly enhanced peripheral nerve localization during surgical procedures. Techniques such as optical molecular imaging and multispectral optoacoustic imaging have emerged, allowing for real-time visualization of nerves. Optical molecular imaging enables precise identification of surgical targets and nerve pathways through color imaging, which is more effective than traditional imaging methods. Similarly, multispectral optoacoustic imaging can visualize peripheral nerves' vascular environment and morphology, providing detailed insights into nerve structure and function. These innovations are crucial for improving the accuracy of nerve blocks, ultimately leading to better patient outcomes [25]. Ultrasound technology has also seen remarkable progress, becoming a standard in clinical practice for the dynamic localization of peripheral nerves based on anatomical landmarks. This method allows for better nerve identification and minimizes the risk of nerve injury during procedures. By leveraging ultrasound guidance, anesthesiologists can accurately target the obturator nerve, enhancing the ONB efficacy while reducing complications. Integrating advanced imaging techniques with traditional practices marks a significant step forward in regional anesthesia [26].

Innovations in ONB techniques are being explored to improve both efficacy and safety. Among these, ultrasound-guided ONB has gained popularity, enabling more accurate localization of the obturator nerve. This method not only enhances the effectiveness of the block but also reduces the likelihood of complications associated with traditional techniques. Recent studies indicate that combining ONB with other imaging modalities, such as MR neurography, can provide comprehensive insights into nerve anatomy and pathology, potentially leading to improved outcomes in nerve blocks and related procedures. This multimodal approach allows for better planning and execution, ensuring that anesthetic agents are delivered precisely where needed [12]. Developing new needle designs and injection techniques also contributes to the refinement of ONB. Using blunt-tipped needles and specialized catheters can facilitate safer and more effective nerve blocks. These innovations underscore the importance of continuous improvement in anesthetic techniques to enhance patient safety and comfort during procedures [27]. The potential for combining ONB with other modalities is particularly promising. Integrating ultrasound guidance with ONB can significantly enhance the precision of nerve localization, thereby reducing the likelihood of complications such as hematoma and nerve injury. Furthermore, optical imaging technologies could refine the identification of nerve pathways during ONB, allowing for real-time adjustments based on intraoperative findings. This ability to visualize nerves dynamically can lead to more effective and safer anesthesia practices [28]. Moreover, combining ONB with multispectral imaging could facilitate a more comprehensive understanding of nerve vascularization and morphology. This knowledge is crucial for optimizing nerve block techniques and improving patient outcomes. By employing advanced imaging technologies alongside traditional nerve block methods, healthcare providers can enhance surgical efficacy and patient safety. This integrative approach highlights the importance of innovation in regional anesthesia, paving the way for improved practices and patient care [29].

Practical recommendations

Implementing ONB in clinical practice for TURBT requires a multifaceted approach. Clinicians should develop evidence-based protocols that incorporate the latest systematic reviews and guidelines. These protocols should standardize techniques, such as favoring ultrasound guidance, which has demonstrated enhanced efficacy and safety. Collaboration among urologists, anesthetists, and nursing staff is crucial to ensuring the seamless integration of ONB into the surgical workflow. Regular monitoring and evaluation of ONB effectiveness in reducing complications during TURBT are essential for refining techniques and improving clinical practice [3]. Clinicians performing ONB should undergo structured training programs encompassing theoretical knowledge and practical skills. These programs should cover anatomy, technique, and potential complications. Simulation-based learning can further enhance clinicians' confidence and proficiency before performing the procedure on patients. Continuing medical education through workshops, seminars, and online courses focused on advancements in anesthetic techniques, including ONB, is crucial for keeping clinicians updated on the latest evidence and techniques. Mentorship programs, where experienced clinicians supervise and guide less experienced colleagues during their initial ONB procedures, can improve skill acquisition and patient safety [30]. Patient selection criteria are paramount when implementing ONB in clinical practice. Prioritizing patients with tumors located near the lateral bladder wall, where the risk of obturator nerve reflex is higher, is critical for maximizing the benefits of ONB. Assessing patients' overall health and comorbidities is essential to determining their suitability for ONB, as certain contraindications may preclude using this technique. Patient preferences regarding anesthesia options should be considered, and informed consent should include discussions about the benefits and risks of ONB versus other anesthesia techniques. Evaluating patients' previous surgical histories, particularly any complications related to anesthesia or bladder surgery, may also influence the decision to perform ONB [3].

## Conclusions

In conclusion, the use of ONB in TURBT represents a significant advancement in enhancing surgical outcomes and patient safety. ONB effectively mitigates the obturator reflex, which is a critical concern during TURBT, by providing targeted anesthesia that prevents involuntary adductor muscle contractions. This technique not only improves the precision of tumor resection but also reduces the risk of complications such as bladder perforation. Comparative studies have demonstrated that ONB offers distinct advantages over other anesthesia methods, including general and spinal anesthesia, particularly in managing intraoperative muscle spasms. While ONB is generally safe, awareness of potential complications and adherence to best practices are essential to minimizing risks. As the field of anesthesiology continues to evolve, further research and innovations in ONB techniques and technologies will likely enhance its efficacy and safety. Overall, incorporating ONB into the anesthesia regimen for TURBT can lead to better surgical conditions, improved patient outcomes, and a higher standard of care in the management of bladder cancer.
